# Supplementary material for: American Dental Association and American Academy of Oral and Maxillofacial Radiology patient selection for dental radiography and cone-beam computed tomography: Clinical recommendations
Source: J Am Dent Assoc. Author manuscript; Available in PMC 2026 Sep 16. (PMC13580382; doi:10.1016/j.adaj.2025.10.013)
Supplement: Supplementary file 1 [file NIHMS2209137-supplement-1.pdf]

APPENDIX 1 - Indication and recall search strategies

Ovid MEDLINE search strategy

1	Exp [explode] Radiography, Dental/
2	((radiograph$ or x-ray$ or radiation or radiology or radiolucency or radiopacity or radiopaque or radiolucent or imaging or bitewing or CBCT [cone-beam computed tomography] or "Cone-beam CT [computed tomography]" or "cone beam computed tomography" or "Computerized tomography" or panoramic or orthopantomograph$) adj5 (dent$ or tooth or teeth or orthodont$ or mouth or maxillofacial or endodont$ or periodont$ or root or maxillary or gingiv$ or intraoral or periapical or alveolar or molar or premolar or cuspid or incisor or canine or temporomandibular or furcation or 'intrabony defect' or 'dental caries' or 'carious lesion')).ab,kw,ti. [abstract, key word, title]
3	1 or 2
4	exp Time Factors/
5	(frequency or frequent or frequently or rate or often or recall or periodically or "prescribing practices" or "time interval" or "time intervals" or timing or routine or ALADA [as low as diagnostically acceptable] or "as low as reasonably achievable" or ALARA). ab,kw,ti.
6	4 or 5
7	(indication$ or indicated or prescribe or prescribed or prescribing or prescription or "selection criteria").ab,kw,ti.
8	6 or 7
9	3 and 8
10	Meta-Analysis as Topic/
11	meta analy$.tw. [text word]
12	metaanaly$.tw.
13	Meta-Analysis/
14	(systematic adj (review$1 or overview$1)).tw.
15	exp "Review Literature as Topic"/
16	review.pt. [publication type]
17	10 or 11 or 12 or 13 or 14 or 15 or 16
18	cochrane.ab. [abstract]
19	embase.ab.
20	(psychlit or psyclit).ab.
21	(psychinfo or psycinfo).ab.
22	(cinahl or cinhal).ab.
23	science citation index.ab.
24	bids.ab.
25	cancerlit.ab.
26	18 or 19 or 20 or 21 or 22 or 23 or 24 or 25
27	reference list$.ab.
28	bibliograph$.ab.
29	hand-search$.ab.
30	relevant journals.ab.
31	manual search$.ab.
32	27 or 28 or 29 or 30 or 31
33	exp guideline/
34	(guideline or guidelines).ab,kw,ot [original title],ti.
35	('consensus statement' or 'consensus statements').ab,kw,ot,ti.
36	33 or 34 or 35
37	selection criteria.ab.

38	data extraction.ab.
39	37 or 38
40	"Review"/
41	39 and 40
42	Comment/
43	Letter/
44	Editorial/
45	exp Animals/
46	exp Humans/
47	45 and 46
48	45 not 47
49	42 or 43 or 44 or 48
50	17 or 26 or 32 or 36 or 41
51	50 not 49
52	9 and 51

Embase search strategy

1	'dental x ray system'/exp
2	'dental radiology'/exp
3	((dent* OR tooth OR teeth OR orthodont* OR mouth OR maxillofacial OR endodont* OR periodont* OR root OR maxillary OR gingiv* OR intraoral OR periapical OR alveolar OR molar OR premolar OR cuspid OR incisor OR canine OR temporomandibular OR furcation OR 'intrabony defect' OR 'dental caries' OR 'carious lesion') NEAR/5 (radiograph* OR 'x ray*' OR radiation OR radiology OR radiolucency OR radiopacity OR radiopaque OR radiolucent OR imaging OR bitewing OR cbct OR 'cone-beam ct' OR 'cone beam computed tomography' OR 'computerized tomography' OR panoramic OR orthopantomograph*)):ab,ti,kw [abstract, title, key word]
4	#1 OR #2 OR #3
5	'time factor'/exp
6	frequency:ti,ab,kw OR frequent:ti,ab,kw OR frequently:ti,ab,kw OR rate:ti,ab,kw OR often:ti,ab,kw OR recall:ti,ab,kw OR periodically:ti,ab,kw OR 'prescribing practices':ti,ab,kw OR 'time interval':ti,ab,kw OR 'time intervals':ti,ab,kw OR timing:ti,ab,kw OR routine:ti,ab,kw OR alada:ti,ab,kw OR 'as low as reasonably achievable':ti,ab,kw OR alara:ti,ab,kw
7	#5 OR #6
8	indication*:ti,ab,kw OR indicated:ti,ab,kw OR prescribe:ti,ab,kw OR prescribed:ti,ab,kw OR prescribing:ti,ab,kw OR prescription:ti,ab,kw OR 'selection criteria':ti,ab,kw
9	'meta analysis'/exp OR 'review'/exp OR 'review':it [item type] OR 'systematic review'/exp OR 'systematic review':it
10	(meta NEXT/1 analy*) OR metaanalys*
11	systematic* NEAR/5 (review* OR overview*)
12	#9 OR #10 OR #11
13	guideline:ti,ab,kw OR guidelines:ti,ab,kw
14	'practice guideline'/exp
15	'consensus statement':ti,ab,kw OR 'consensus statements':ti,ab,kw
16	#13 OR #14 OR #15
17	'cancerlit':ab
18	'cochrane':ab
19	'embase':ab
20	'psychlit':ab OR 'psyclit':ab
21	'psychinfo':ab OR 'psycinfo':ab

22	'cinahl':ab OR 'cinhal':ab
23	'science citation index':ab
24	'bids':ab
25	#17 OR #18 OR #19 OR #20 OR #21 OR #22 OR #23 OR #24
26	'reference lists':ab
27	'bibliograph*':ab
28	'hand-search*':ab
29	'manual search*':ab
30	'relevant journals':ab
31	#26 OR #27 OR #28 OR #29 OR #30
32	'letter':it
33	'editorial':it
34	'animal'/exp
35	'human'/exp
36	#34 NOT (#34 AND #35)
37	#32 OR #33 OR #36
38	#12 OR #16 OR #25 OR #31
39	#38 NOT #37
40	#7 OR #8
41	#4 AND #40
42	#39 AND #41

Cochrane Database of Systematic Reviews search strategy

1	MeSH [Medical Subject Heading] descriptor: [Radiography, Dental] explode all trees
2	((radiograph* OR x-ray* OR radiation OR radiology OR radiolucency OR radiopacity OR radiopaque OR radiolucent OR imaging OR bitewing OR CBCT OR "Cone-beam CT" OR "cone beam computed tomography" OR "Computerized tomography" OR panoramic OR orthopantomograph*) NEAR/5 (dent* OR tooth OR teeth OR orthodont* OR mouth OR maxillofacial OR endodont* OR periodont* OR root OR maxillary OR gingiv* OR intraoral OR periapical OR alveolar OR molar OR premolar OR cuspid OR incisor OR canine OR temporomandibular OR furcation OR 'intrabony defect' OR 'dental caries' OR 'carious lesion')):ti, ab,kw (Word variations have been searched)
3	#1 OR #2
4	MeSH descriptor: [Time Factors] explode all trees
5	(frequency OR frequent OR frequently OR rate OR often OR recall OR periodically OR "prescribing practices" OR "time interval" OR "time intervals" OR timing OR routine OR ALADA OR "as low as reasonably achievable" OR ALARA):ti,ab,kw (Word variations have been searched)
6	#4 OR #5
7	(indication* OR indicated OR prescribe OR prescribed OR prescribing OR prescription OR "selection criteria"):ti,ab,kw
8	#6 OR #7

APPENDIX 2 - Nine clinical questions for radiography update to the 2012 recommendations[3]

1	What radiologic evaluation is recommended during patient recall visits for patients with clinical caries or based on professional opinion are at increased risk of developing caries?
2	What radiologic evaluation is recommended during patient recall visits for patients with no caries or based on professional opinion do not seem to be at increased risk of developing caries?

3	What radiologic evaluation is recommended for new patients?
4	What radiologic evaluation is recommended during patient recall visits for patients with periodontal disease?
5	What radiologic evaluation is recommended to assess dentofacial development?
6	What are positive clinical signs or symptoms for clinical situations in which radiologic evaluation may be indicated?
7	Which radiologic evaluations are appropriate for planning, intraoperative, and follow-up evaluation for implants?
8	Which radiologic evaluations are appropriate for diagnosis and intraoperative and follow-up evaluation for endodontic lesions?
9	What radiologic evaluation is recommended for assessment of dental trauma?

e1. Page MJ, McKenzie JE, Bossuyt PM, et al. The PRISMA 2020 statement: an updated guideline for reporting systematic reviews. *BMJ*. 2021;372:n71. doi:10.1136/bmj.n71

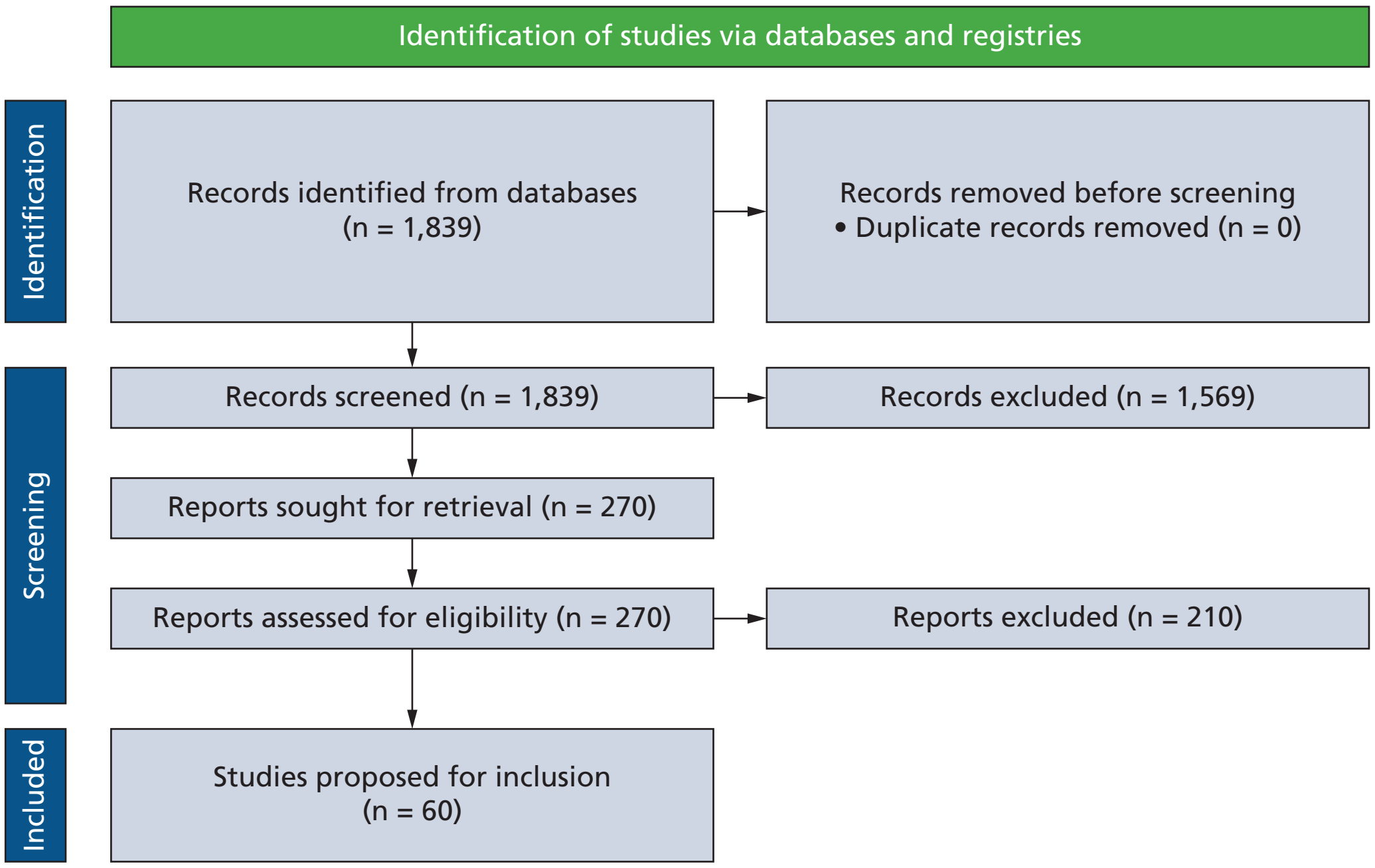

eFigure. Preferred Reporting Items for Systematic Reviews and Meta-Analyses flow diagram.[e1]
